# Comprehensive Functional Annotation of 77 Prostate Cancer Risk Loci

**DOI:** 10.1371/journal.pgen.1004102

**Published:** 2014-01-30

**Authors:** Dennis J. Hazelett, Suhn Kyong Rhie, Malaina Gaddis, Chunli Yan, Daniel L. Lakeland, Simon G. Coetzee, Ellipse/GAME-ON consortium, Practical consortium, Brian E. Henderson, Houtan Noushmehr, Wendy Cozen, Zsofia Kote-Jarai, Rosalind A. Eeles, Douglas F. Easton, Christopher A. Haiman, Wange Lu, Peggy J. Farnham, Gerhard A. Coetzee

**Affiliations:** 1 Departments of Urology and Preventive Medicine, Norris Cancer Center, University of Southern California Keck School of Medicine, Los Angeles, California, United States of America; 2 Department of Biochemistry and Molecular Biology, Keck School of Medicine, University of Southern California, Los Angeles, California, United States of America; 3 Sonny Astani Department of Civil and Environmental Engineering, University of Southern California, Los Angeles, California, United States of America; 4 Department of Genetics, University of São Paulo, Ribeirão Preto, Brazil; 5 Department of Preventive Medicine, Norris Cancer Center, University of Southern California Keck School of Medicine, Los Angeles, California, United States of America; 6 The Institute of Cancer Research, Sutton, United Kingdom; 7 USC Keck School of Medicine, Norris Comprehensive Cancer Center, University of Southern California, Los Angeles, California, United States of America; 8 Royal Marsden National Health Service (NHS) Foundation Trust, London and Sutton, United Kingdom; 9 Centre for Cancer Genetic Epidemiology, Department of Oncology, University of Cambridge, Cambridge, United Kingdom; 10 Eli and Edythe Broad Center for Regenerative Medicine and Stem Cell Research, Department of Biochemistry and Molecular Biology, Keck School of Medicine, University of Southern California, Los Angeles, California, United States of America; University of Michigan, United States of America

## Abstract

Genome-wide association studies (GWAS) have revolutionized the field of cancer genetics, but the causal links between increased genetic risk and onset/progression of disease processes remain to be identified. Here we report the first step in such an endeavor for prostate cancer. We provide a comprehensive annotation of the 77 known risk loci, based upon highly correlated variants in biologically relevant chromatin annotations— we identified 727 such potentially functional SNPs. We also provide a detailed account of possible protein disruption, microRNA target sequence disruption and regulatory response element disruption of all correlated SNPs at 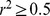. 88% of the 727 SNPs fall within putative enhancers, and many alter critical residues in the response elements of transcription factors known to be involved in prostate biology. We define as risk enhancers those regions with enhancer chromatin biofeatures in prostate-derived cell lines with prostate-cancer correlated SNPs. To aid the identification of these enhancers, we performed genomewide ChIP-seq for H3K27-acetylation, a mark of actively engaged enhancers, as well as the transcription factor TCF7L2. We analyzed in depth three variants in risk enhancers, two of which show significantly altered androgen sensitivity in LNCaP cells. This includes rs4907792, that is in linkage disequilibrium (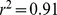) with an eQTL for NUDT11 (on the X chromosome) in prostate tissue, and rs10486567, the index SNP in intron 3 of the JAZF1 gene on chromosome 7. Rs4907792 is within a critical residue of a strong consensus androgen response element that is interrupted in the protective allele, resulting in a 56% decrease in its androgen sensitivity, whereas rs10486567 affects both NKX3-1 and FOXA-AR motifs where the risk allele results in a 39% increase in basal activity and a 28% fold-increase in androgen stimulated enhancer activity. Identification of such enhancer variants and their potential target genes represents a preliminary step in connecting risk to disease process.

## Author Summary

In the following work we provide a complete summary annotation of functional hypotheses relating to risk identified by genome wide association studies of prostate cancer. In addition, we present new genome-wide profiles for H3K27-acetylation and TCF7L2 binding in LNCaP cells. We also introduce the concept of a risk enhancer, and characterize two novel androgen-sensitive enhancers whose activity is specifically affected by prostate-cancer risk SNPs. Our findings represent a preliminary approach to systematic identification of causal variation underlying cancer risk in the prostate.

## Introduction

The basic goal of research into human genetics is to connect variation at the genetic level with variation in organismal and cellular phenotype. Until recently, inferences about such connections have been limited to the kind associated with heritable disorders and developmental syndromes. Such variations often turn out to be the result of disruptions to protein coding sequences of critical enzymes for an affected pathway. Recent advances in genomics and medicine have begun to illuminate a sea of variation of a more subtle variety, not always the result of mutation of protein coding sequences. In particular, genome-wide association studies (GWAS) have identified thousands of variants associated with hundreds of disease traits [1]. These variants, typically encoded by single nucleotide polymorphisms (SNPs), are given landmark status and called ‘index-SNPs’ (they are also frequently referred to in the literature as ‘tag-SNPs’) as the reference for disease or phenotype association in that region. The vast majority of these variants reside within intergenic or intronic regions [2], prompting at least two new avenues of inquiry: 1) What is the nature and scope of risk encoded at these ‘non-coding’ loci?, and 2) What are the target genes, and how do these alterations account for increased risk in a disease?

At present, little is known regarding the functional mechanisms of the common variant susceptibility loci in non-coding regions. For one, there are many genetically correlated variants that—to varying degrees—may account for the risk associated with each index-SNP. It is unclear whether more than one variant carries functional consequences relevant to the risk that was reported. In addition, we are only beginning to understand the nature of non-coding regions as revealed by histone modifications and other chemical signatures on chromatin. Efforts to fill this void are underway, notably by the ENCODE consortium [3], whose goal it is to catalog all the major chromatin biofeatures, including histone modifications, accessible chromatin and transcription factor bound regions in the form of digital footprinting and ChIP-seq for transcription factors, among others. Currently, a mosaic of annotations for all the known histone modifications and 119 different transcription factors has been released for 147 cell types, including an androgen-sensitive prostate adenocarcinoma cell line isolated from lymph-node metastasis, called Lymph Node Cancer of the Prostate (LNCaP) [4]–[6]. Insights into cancer biology of the prostate have already begun to emerge from this work. For example, risk polymorphisms for the 8q24 locus have been extensively characterized in our lab and others [7], [8].

We propose that by identifying all the variants that are in linkage disequilibrium with GWAS SNPs and subsequently filtering down to those present within genome-wide functional annotations we will identify the most likely causal susceptibility variants within regulatory elements that can be tested for their functional significance. We previously developed the R-Bioconductor package *Funci–SNP* *}* [2] which performs these operations, including the linkage disequilibrium calculations, based on data from the 1,000 genomes project (www.1000genomes.org [9]) automatically. With the advent of *Funci–SNP*} and similar tools such as RegulomeDB [10], performing annotations of this type becomes possible, and indeed essential to understanding the candidate variations that may underlie risk for disease.

Post-GWAS analyses of breast cancer [11] for example identified putative functional variants using *Funci{SNP*} and genome-wide chromatin biofeature data for breast epithelia-derived cell lines as described above, but this level of detail is lacking for prostate cancer. In that study, we catalogued and assessed the correlated functional variants at 72 breast cancer risk loci and performed preliminary enrichment analysis of motifs. We identified over 1,000 putative functional SNPs, most of which were in putative enhancers. We provide here a similar analysis for prostate cancer, extending the previous work and introducing some improvements to the downstream analyses. We also present some new ChIP-seq datasets to add to ENCODE.

## Results

### Classification of variants associated with prostate cancer

In order to identify variants that are in linkage disequilibrium with 77 prostate cancer risk loci (defined as all significant GWAS, replication study and post-GWAS identified variants, see Table 1 for references), that are also relevant to the biology of prostate epithelia, we employed our bioinformatics tool, *Funci*{*SNP*} [2] to integrate biofeatures with 1000 genomes data [9] (see Methods for a detailed list of biofeatures). For the LNCaP cell line, genome-wide data are generally available both with and without androgen treatment. Since the androgen receptor is a driver of prostate cancer [12], we included both conditions where possible. We also considered protein coding exons, 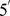 and 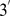 untranslated regions with miRcode target sequences. Importantly, we also included the index-SNPs in our analysis.

**Table 1:** Independent risk loci.

Locus	genomic position	SNP	Gene	Ethn
1	1q32.1	rs4245739 [19]	MDM4	AFR
2	2p24.1	rs13385191 [30], [32]	C2orf43	EUR
3	2p21	rs1465618 [28]	THADA	EUR
4	2p15	rs6545977 [19], [28]	EHBP1	AFR, EUR
5	2p15	rs721048 [77]	EHBP1, OTX1	EUR
6	2p11.2	rs10187424 [33]	GGCX	EUR
7	2q31.1	rs12621278 [19], [28]	ITGA6	AFR+EUR
8	2q37.3(*cont'd*)	rs2292884 [33],rs7584330 [19]	MPLH…	EURAFR+EUR
9	3p22.2	rs9311171 [78]	CTDSPL	EUR
10	3p12.1-2	rs17181170 [28]	CHMP2B	EUR
11	3p12.1-2	rs2660753 [27], rs9284813 [30], [32]	CHMP2B	EUR
12	3p12.1-2	rs7629490 [33]	CHMP2B	EUR
13	3q21.3	rs10934853 [29]	GATA2	EUR
14	3q23	rs6763931 [33]	ZBTB38	EUR
15	3q24	rs345013 [78]	PLOD2	EUR
16	3q26.2	rs10936632 [33]	CLDN11, SKIL	EUR
17	4q22.3	rs17021918 [19], [28]	PDLIM5	AFR+EUR
18	4q22.3	rs12500426 [28]	PDLIM5	EUR
19	4q24	rs7679673 [19], [28]	TET2	AFR+EUR
20	5p15.33	rs2242652 [28]	TERT	EUR
21	5p15.33	rs12653946 [19], [30], [32]	LPCAT1	AFR+EUR
22	5p12	rs2121875 [33]	FGF10	EUR
23	5q14.3	rs4466137 [78]	HAPLN1	EUR
24	5q23.1	rs37181 [33]	COMMD10	EUR
25	6p21.1	rs1983891 [19], [30], [32]	FOXP4	AFR+EUR
26	6p12.2	rs10498792 [78]	PKHD1	EUR
27	6q22.2	rs339331 [19], [30], [32]	RFX6	AFR+EUR
28	6q25.3	rs651164 [28], [33]	IGF2R	EUR
29	6q25.3	rs9364554 [19], [27]	SLC22A3	AFR+EUR
30	7p15.3	rs12155172 [28]	RPL23P8	EUR
31	7p15.2	rs10486567 [19], [26]	JAZF1	AFR+EUR
32	7q21.3	rs6465657 [27], [28]	LMTK2	AFR+EUR
33	8p21.2	rs1512268 [19], [28], [30], [32]	NKX3-1	AFR+EUR
34	8q24.21	rs12543663 [19]	LOC727677, MYC	AFR
35	8q24.21	rs10086908 [27]	POU5F1B, MYC	EUR
36	8q24.21	rs1016343 [27], [33]	POU5F1B, MYC	EUR
37	8q24.21	rs13252298 [19], [33]	PCAT1, MYC	AFR+EUR
38	8q24.21(*cont'd*)	rs1456315 [30], [32],rs13254738 [19]	PCAT1, MYC…	EURAFR
39	8q24.21(*cont'd*)	rs6983561 [19],	PCAT1, MYC…	AFREUR
40	8q24.21	rs188140481 [34]	PCAT1, MYC	EUR
41	8q24.21	rs16902094 [29]	PCAT1, MYC	EUR
42	8q24.21	rs445114 [29], [33]	PCAT1, MYC	EUR
43	8q24.21	rs6983267 [19], [24], [26], [27], [33]	PCAT1, MYC	AFR+EUR
44	8q24.21	rs7000448 [19], [31]	LOC727677, MYC	AFR+EUR
45	8q24.21(*cont'd*)……	rs1447295 [24], [25], [29],rs4242382 [26],rs4242384 [27], [28], [33],rs7837688 [30], [32]	POU5F1B, MYC………	EUR…EUR…
46	9q31.2	rs817826 [19], [79]	KLF4	ASN+AFR
47	9q33.2	rs1571801 [80], [81]	DAB2IP	ASN+EUR
48	10q11.23(*cont'd*)	rs10993994, [19], [26], [27], [30],[32],[33]rs3123078 [28]	NCOA4…	AFR+EUREUR
49	10q26.12	rs11199874 [82]	FGFR2	EUR
50	10q26.13	rs4962416 [26]	CTBP2	EUR
51	11p15.5	rs7127900 [19], [28]	IGF2	AFR+EUR
52	11q13.2(*cont'd*)	rs10896449 [19], [26],rs7931342 [27]	CCND1…	AFR+EUREUR
53	11q13.2	rs12418451 [83]	CCND1	EUR
54	11q13.2(*cont'd*)	rs11228565 [19], [29],rs7130881 [28], [33]	CCND1…	AFR+EUREUR
55	12q13.12	rs731236 [84]	VDR	EUR
56	12q13.13	rs10875943 [33]	TUBA1C	EUR
57	12q13.2	rs902774 [33]	KRT8	EUR
58	12q21.31	rs12827748 [84]	PAWR	EUR
59	13q22.1	rs9600079 [30], [32]	KLF5	EUR
60	13q33.2	rs1529276 [78]	MIR548AS	EUR
61	15q21.1	rs4775302 [82]	SQRDL	EUR
62	17p13.3	rs684232 [19]	VPS53	AFR
63	17q21.2(*cont'd*)	rs7501939 [27], [28], [30], [32], [33],rs4430796 [26], [29], [85], [86]	HNF1B…	EUR…
64	17q21.2	rs11649743 [86]	HNF1B	EUR
65	17q21.33	rs138213197 [34], [45]	HOXB13	EUR
66	17q21.33	rs11650494 [19]	ZNF652	AFR
67	17q21.33	rs7210100 [87]	ZNF652	EUR
68	17q25.1	rs1859962 [27], [28], [33], [85]	BC039327	EUR
69	19q13.4	rs103294 [79]	LILRA3	ASN
70	19q13.11	rs8102476 [19], [29]	SPINT2	AFR+EUR
71	19q13.12	rs887391 [88]	LOC100505495	EUR
72	19q13.32	rs2735839 [27]	KLK3	EUR
73	22q13.1	rs9623117 [89]	TNRC6B	EUR
74	22q13.2	rs742134 [33]	PACSIN2	EUR
75	22q13.2	rs5759167 [19], [28]	BIK	AFR+EUR
76	Xp11.22(*cont'd*)	rs5945572 [19], [77],rs5945619 [27], rs1327301 [28]	NUDT11…	AFR+EUREUR
77	Xq12	rs5919432 [19], [33]	AR	AFR+EUR

**Independent GWAS Loci.** Table of independent associations with prostate cancer. Index SNPs with 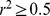 are grouped together, and shown with source citations. A locus with a significant number of correlated SNPs at 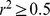 for two index SNPs that don't meet the cutoff are also considered the same locus. Also shown are the nearby genes (Gene) and population in which the associations were reported (Ethn).

**Figure 1:**
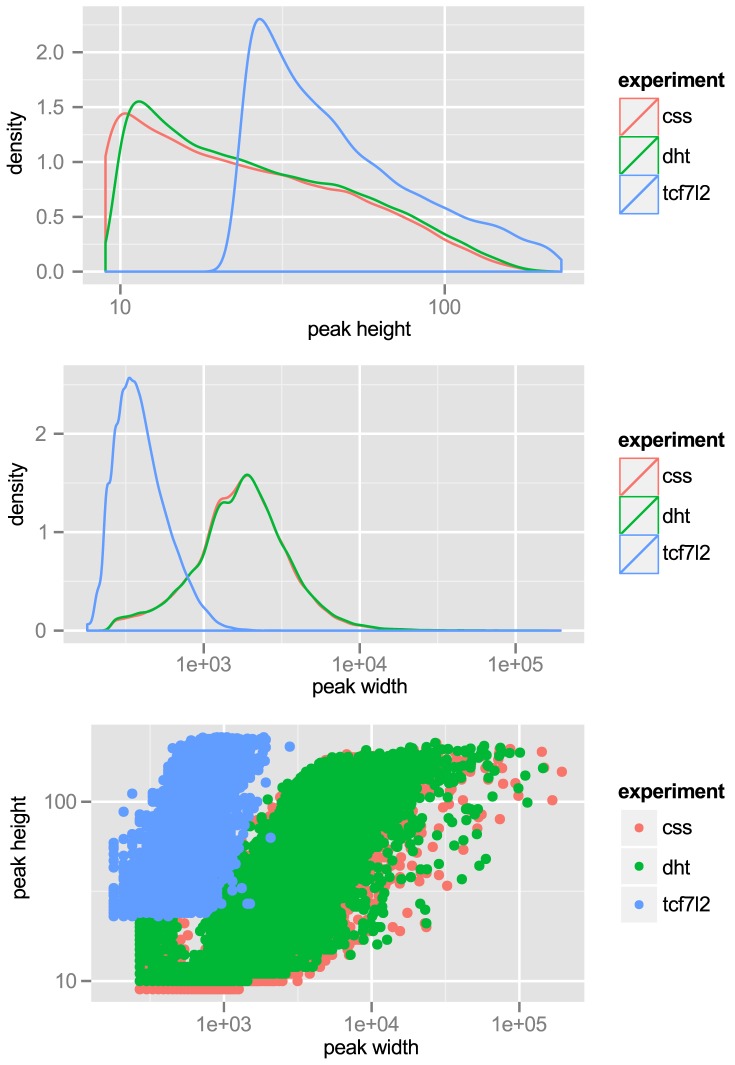
Tag-density profiles of ChIP-seq datasets ‘css’: H3K27Ac ChIP-seq of LNCaP grown in charcoal-stripped serum. ‘dht’: H3K27Ac ChIP-seq of LNCaP exposed to androgen. ‘tcf7l2’: ChIP-seq with anti-TCF7L2 in LNCaP, unstimulated. Top: peak height, 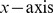 is 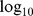 scaled. Middle: peak width, 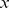-axis is 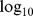 scaled. Bottom: Peak height *vs.* width reveals strong correlation.

We note that some critical datasets were not available when we initiated our studies. For example, ChIP-seq data for the histone modification H3K27Ac was not available for LNCaP cells. This is a mark of active enhancers, which are extremely cell-type specific. Although other marks, such as DNase I hypersensitivity or H3K4me1, can reveal regions of open chromatin, they do not identify active enhancers. Therefore, we performed ChIP-seq for H3K27Ac in LNCaP cells, after a period of incubation in charcoal-stripped serum (*i.e.* androgen depleted) followed by exposure to vehicle control or physiological levels of the androgen dihydrotestosterone (10 nM DHT). For LNCaP treated with vehicle (minus DHT) we observed 57,623 peaks, with an average peak height of 32 tags and median height of 22 tags, and a range of 9 to 212 tags. The average peak width was 2,233 bp. For LNCaP post-androgen stimulation, we observed 60,752 peaks, with an average peak width of 2,267 bp. Overall the relative tag density and peak width distribution was extremely similar between the two conditions (see Figure 1, top and middle panels). A plot of peak height *vs.* peak width reveals a linear relationship in log space (Figure 1, bottom panel). Because we wanted to limit our studies to robust enhancers, we chose the top 25,000 peaks, which have a tag density of 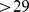 for use in *Funci*{*SNP*}. This cutoff marks an inflection point where the number of tags increases geometrically over background (Figure S1). A comparison of the top 25,000 H3K27Ac peaks detected before and after induction with DHT revealed an 84% overlap (see Figure S2), suggesting that only a small percentage of all H3K27Ac peaks are responsive to hormone treatment.

We also wished to include transcription factor binding data in our analyses. Although there were data available for ChIP-seq of androgen receptor (AR), FOXA1 and NKX3-1, data for TCF7L2— another transcription factor with a proposed role in prostate- and other cancers [13]— was not available. Therefore we performed ChIP-seq for TCF7L2 in LNCaP. We chose the top 15,000 peaks, with an average peak height of 57 tags and a range of 23 to 229 tags and an average peak width of 432 bp. These properties are also displayed graphically in Figure 1. TCF7L2 binding sites were also highly enriched in the center of TCF7L2 ChIP-seq peaks (Figure S3).

**Figure 2:**
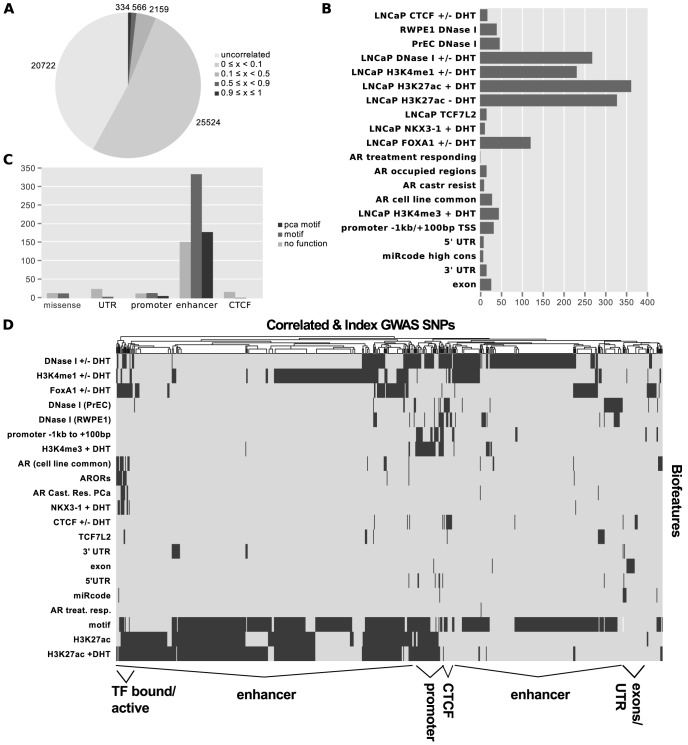
Results of *Funci{SNP}* analysis of GWAS correlated SNPs. Index SNPs with biofeatures and correlated SNPs at 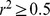 are combined and summarized in A–D. A. SNP counts by 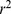 value. B. SNP counts by biofeature. Some SNPs map to more than one biofeature, hence the total does not sum to 727. C. Classification of 727 SNPs by *putative* functional category. D. Supervised clustering of SNPs by biofeature.

Using *Funci*{*SNP*}, we identified 49,305 SNPs that were correlated in the population in which the original index SNP was reported within prostate epithelial chromatin biofeatures, of which only 727 had an 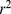 value greater than or equal to 0.5 (Figure 2A). The most common SNP annotations are associated with H3K27-acetylation (385 SNPs) and the other enhancer marks H3K4-monomethylation (231 SNPs) and LNCaP DNaseI hypersensitivity (268 SNPs, see Figure 2B). A complete visualisation of correlated SNPs with 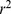 and all associated biofeatures are available on the UCSC genome browser; furthermore all custom tracks may be downloaded in bed format via the table browser therein: http://genome.ucsc.edu/cgi-bin/hgTracks?hgS_doOtherUser=submit&hgS_otherUserName=hazelett&hgS_otherUserSessionName=pca.

After identifying SNPs in primary biofeatures, we grouped them according to putative functional classes for further analysis. We identified 30 SNPs in putative promoter regions −1000 bp to +100 bp relative to transcription start sites, 663 SNPs in putative enhancer regions, 4 SNPs in microRNA target sequences within 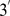 or 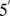 UTRs, and 27 SNPs in coding exons (Figure 2C).

To directly observe the relationships of the annotations to each SNP across the entire set, we performed unsupervised clustering on the matrix of biofeatures and SNPs (Figure 2D). The resulting cluster diagram neatly captures the functional categories, but also reveals a cluster of SNPs in regions bound by multiple transcription factors. Perhaps most importantly, Figures 2C and 2D clearly show that the majority of variation associated with risk for prostate cancer resides within what we have defined as putative risk enhancers.

### Functional annotation of exon variants

We identified 27 exon SNPs in linkage disequilibrium with index SNPs for prostate cancer (Figure 2B & 2C). Of these SNPs, 13 encoded missense substitutions in coding exons, 14 encoded synonymous substitutions, and 0 corresponded to nonsense condons or other types of lesions (Table 2). We conducted a preliminary exploration of the potential effects of the 11 missense variants using publically available software packages PROVEAN [14], SIFT [15], Polyphen2 [16], and SNAP [17]. The results of this analysis are summarized in Table 2. All four algorithms predicted that a single index-SNP, the rare variant rs138213197, encoding a Glycine to Glutamine substitution at position 84 of the homeobox transcription factor HOXB13, has a deleterious effect. Two other missense variants, rs2452600 (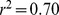) and rs7690296 (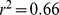), correlated to index SNP rs17021918, encoded potentially damaging changes in the PDLIM5 gene. Three of four algorithms predicted rs2452600 to be damaging or non-neutral, and rs17021918 was only predicted to be non-neutral by SNAP. Three missense variants in the MLPH gene were not predicted to be deleterious, but were highly correlated to each other 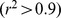 and only weakly correlated to index SNP rs2292884 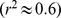, raising the possibility that together they form a haplotype that weakens or damages protein function.

**Table 2:** Missense variants in correlated SNPs.

snp	gene	AA	PROVEAN	SIFT	Polyphen2	SNAP
rs11765552	LMTK2	L780M			Possibly damaging	Non-neutral
rs2274911	GPCR6A	P91S	Deleterious			Non-neutral
rs6998061	POU5F1B	G176E	Deleterious			
rs5995794	FAM83F	R436G		Damaging		Non-neutral
rs383369	LILRB2	H20R	Deleterious			
rs386056	LILRB2	V235M				
rs3751107	MLPH	G172D				
rs3751109	MLPH	L153P				
rs11883500	MLPH	T289I				
rs2292884	MLPH	H347R				
rs2452600	PDLIM5	S136F		Damaging	Possibly damaging	Non-neutral
rs7690206	PDLIM5	T410A				Non-neutral
rs138213197	HOXB13	G84E	Deleterious	Damaging	Probably damaging	Non-neutral

**Non-synonymous substitutions.** Table of *Funci{SNP}*-identified single nucleotide missense variants in protein coding exons, showing the results of variant effect prediction software.

We next identified 29 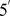 and 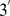 UTR SNPs, of which 4 occur within microRNA target element regions. We cross referenced against highly conserved, high-scoring elements defined by miRcode [18]. Index SNP rs4245739 was located within a miR target sequence in the 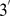 UTR of the *MDM4* gene. This SNP was previously reported in functional annotation of iCOGS [19] for prostate cancer, esophogeal squamous cell carcinoma [20] and is a functional variant in breast cancer [21]. The other three variants affect putative target sequences in the *HAPLN1*, *SLC22A3*, and *FOXP4* genes, and are also of potential interest (see Table 3 for details).

**Table 3:** miR-target variants.

SNP	r2	miR recognition seq	location	gene
rs3734092	0.95	miR-210	5′UTR	HAPLN1
rs1810126	0.59	miR-124/506	3′UTR	SLC22A3
rs4245739	index	miR-191	3′UTR	MDM4
rs6935737	0.91	miR-183	5′UTR	FOXP4

**SNPs in miR target sequences.** Table of SNPs affecting putative miR target sequences in untranslated coding regions, and the potentially affected target genes.

### Annotation of enhancers and putative functional SNPs

**Figure 3:**
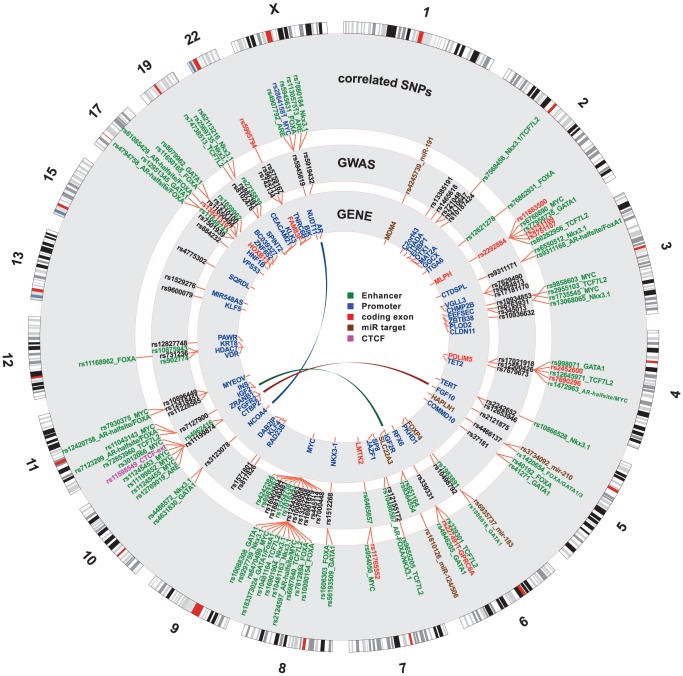
Genome-wide summary of functional annotations. Detailed map of the locations and annotations associated with risk for prostate cancer throughout the human genome. Each ring shows, successive from center, the names and locations of proximal genes, the tag- or index-SNPs, and the correlated 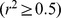 SNPs. The links in the center highlight known biochemical interactors (*e.g.* receptor-ligand pairs). Index and correlated SNPs are color-coded by putative functional category (see Legend, center). Potentially disrupted response elements are also indicated for the correlated SNPs. The outermost ring shows the numbered chromosomes to scale with cytological banding patterns. The genome is displayed clockwise from top, with p displayed as the left arm of each chromosome and q as the right arm.

In order to identify putative functional variants within proposed enhancer and promoter regions, 663 SNPs from enhancers and 30 SNPs from promoters were queried against 87 positional weight matrices (PWM) compiled from Factorbook [22] (see Methods). Factorbook includes response element definition for the FOXA family of transcription factors, TCF7L2, MYC, and GATA1 and -3 among others. In addition we used PWMs from Homer [23] for FOXA1, the androgen receptor (AR) and NKX3-1. We identified a subset of 509 variants in putative enhancers and 20 variants in promoter regions that disrupt response elements (see UCSC genome-browser http://genome.ucsc.edu/cgi-bin/hgTracks?hgS_doOtherUser=submit&hgS_otherUserName=hazelett&hgS_otherUserSessionName=pca). For both promoters and enhancers we also identified a subset of disruptive variants that target response elements for factors of special interest to prostate cancer, namely AR, FOXA1, NKX3-1, TCF7L2, MYC, GATA1 and GATA3. There were 6 SNPs in promoters and 177 in enhancers for this short list of PCa-specific factors. These findings for PCa response elements are summarized in Figure 3.

**Figure 4:**
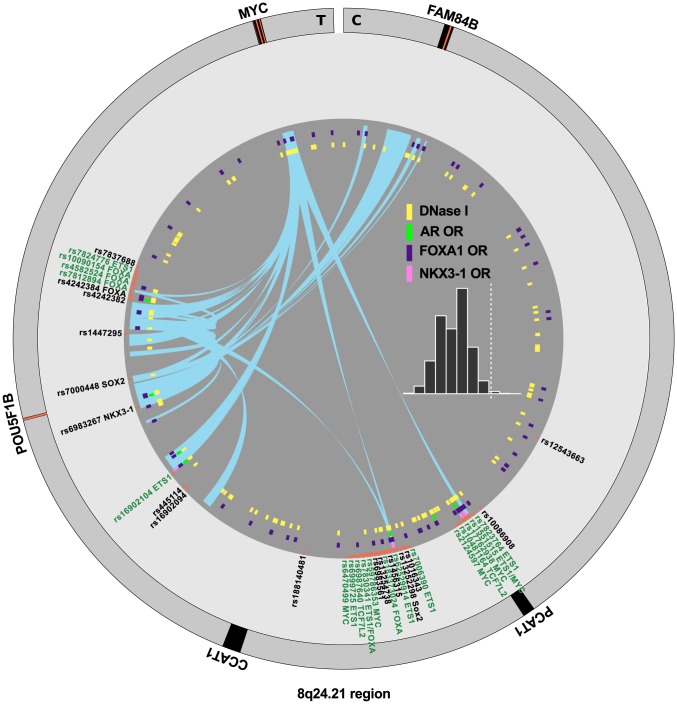
Annotation of the 8q24.21 region. The intergenic region between FAM84B and MYC is shown with biofeatures indicated as colored hashes in the inside tracks. Index SNPs are black, correlated enhancer snps are in green according to the convention in Figure 3. Chromatin capture 5C data are indicated as links (light blue) in the center, showing interactions between regions. Histogram (inset) indicates the distribution of the dataset, showing the tag density on the 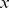-axis *vs.* number of regions. The dotted line indicates *min.* tag-density cutoff for the display.

There are many densely situated independent risk loci in the 8q24.21 region centromeric of the MYC oncogene [19], [24]–[34], which therefore warranted additional consideration. Figure 4 displays the region zoomed in to 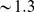 Mb. Because 5C chromatin conformation capture data are available for the 8q24 region in LNCaP through ENCODE [3], we examined the relationship of these data to our risk enhancers. A circos plot showing interacting regions with the highest tag densities (see histogram inset with dotted cutoff in Figure 4) reveals extensive overlap between putative risk enhancers and sites of intrachromasomal interaction. Several SNPs effecting FOXA1 and ETS1 transcription factor binding sites in the vicinity of the POU5F1B locus are located within putative enhancer regions that interact in a complex manner with each other, with the *POU5F1B* coding region, and with both the *MYC* and *FAM84B* genes. Another locus, the *PCAT1* non-coding gene, has several SNPs affecting MYC, ETS1 and TCF7L2 candidate binding sites that potentially interact with the *MYC* gene locus (Figure 4). Another putative enhancer situated between *PCAT1* and *CCAT1* non-coding RNA genes interacts with the enhancer telomeric of *POU5F1B* pseudogene and also with *MYC*. It is striking from this view that 7 of the 16 index SNPs (rs7837688, rs1447295, rs445114, rs16902094, rs188140481, rs10086908, rs12543663) do not overlap any biofeatures or chromatin 5C capture data, whereas the correlated enhancer SNPs with response element disruptions do. These variants cluster within 5C-interacting regions despite having been filtered with LNCaP biofeatures, which are distributed evenly throughout the region (see for example DNase I and FOXA1 tracks in Figure 4). These data are consistent with the hypothesis that some GWAS hits have no direct effect, but instead are correlated to nearby functional variants.

### Definition of risk loci

After the *Funci*{*SNP*} analysis, many index SNPs had redundant associations with correlated SNPs. We examined each locus carefully to determine the number of unique and independent risk loci. Starting from a list of 91 SNPs as input to *Funci{SNP}*, we determined that there were 77 loci that were independent. We tabulated the independent risk loci in sequential order (Table 1) in the genome.

**Figure 5:**
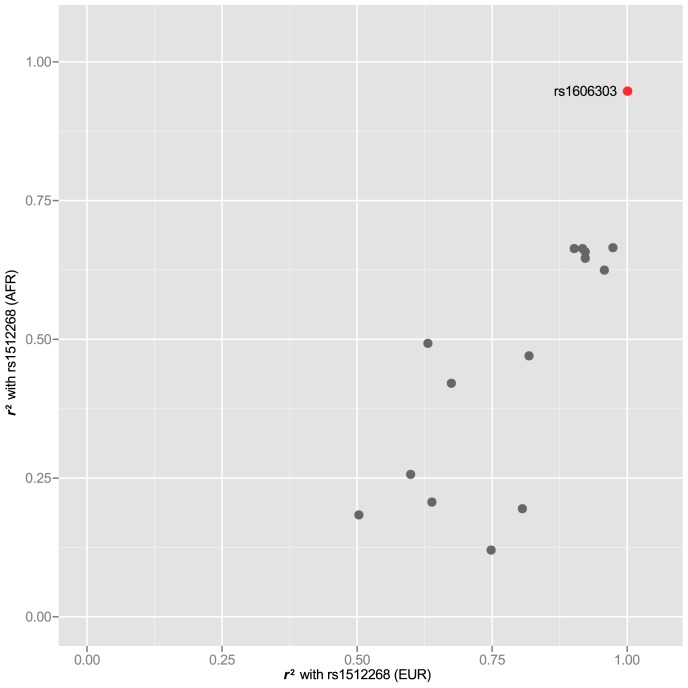
rs1512268 in two populations. The rs1512268 risk locus is 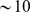 kb downstream of the *NKX3-1* gene. An 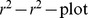 reveals SNPs that are correlated to the index SNP in both populations for which it has been identified as carrying risk. One SNP that is highly correlated in populations of both African and European ancestry is highlighted in red.

In 25 of the 77 risk loci, we also were able to examine the LD structure for index SNPs that have been reported in two ethnic groups. For these SNPs, we asked whether some SNPs had higher correlation with the index SNP in both GWAS-tested populations (see Table 1 for population). For example rs1512268 near the *NKX3-1* gene, which reached genome-wide significance for both African and European populations (see Table 1 for references), was correlated to 15 other SNPs at 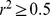, but a single SNP, rs1606303 was highly correlated at 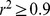 in populations with both African and European ancestry (Figure 5). Thus, we have also identified subsets of SNPs in the supplementary materials for rs12621278 (Figure S4), rs7584330 (Figure S5), rs17021918 (Figure S6), rs7679673 (Figure S7), rs12653946 (Figure S8), rs1983891 (Figure S9), rs339331 (Figure S10), rs9364554 (Figure S11), rs10486567 (Figure S12), rs6983267 (Figure S13), rs7127900 (Figure S14), rs10896449 (Figure S15), rs11228565 (Figure S16) and rs8102476 (Figure S17) present in different ethnic groups.

Nine other loci, at rs2710647, rs6465657, rs13252298, rs7000448, rs817826, rs1571801, rs10993994, rs5759167 and rs5919432 did not have any SNPs at 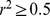 in both populations. It is possible that the likeliest functional SNP in these cases is the index SNP. One remaining SNP, rs5945572 in the NUDT11 region, was identified in African and European populations (see Table 1 for refs.), and also correlated to the same three SNPs as two other index SNPs, rs1327301 and rs5945619. However, rs1327301 and rs5945619, which were identified in Europeans (see Table 1 for refs.) surprisingly were *not* correlated to rs5945572 in Africans. Two of the three correlated SNPs encode disruptions of MYC (rs28641581) and AR (rs4907792, marked for functional followup, see below) binding sites in putative enhancers. Therefore, we hypothesize that all three index SNPs in this region are correlated to these other functional SNPs as the primary source of risk, and that together they constitute a single independent risk locus (#76 in Table 1).

### Motif enrichment

**Figure 6:**
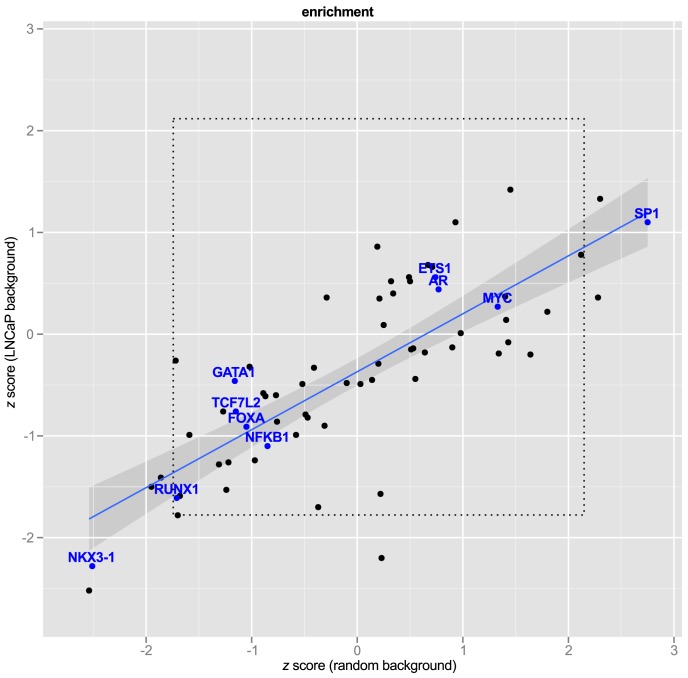
Transcription Factor Response Elements are not enriched in PCa GWAS SNPs. 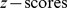
 express number of observed response element disruptions as a proportion relative to the standard deviation from the background distribution. The regression line is shown in blue with 95% confidence interval. Transcription factors of interest are highlighted with blue text. The inner box (dotted line) demarcates the 95% C.I. of a bootstrapped distribution for each PWM. A bonferroni box is outside the bounds of the graphic.

We next asked whether the 663 enhancer SNPs were enriched for disruption in any of the 87 PWMs chosen from Factorbook and Homer. In other words, we wanted to know whether disruption of any specific transcription factor response elements was associated with GWAS SNPs at greater than expected frequency. We approached this question in two ways. First, we asked whether response element disruptions were enriched against a background of randomly selected SNPs. In order to ensure that we were drawing inference from the background distribution we drew samples (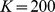) of random SNPs (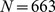), counted the number of motif disruptions for each of the 87 factors, and bootstrapped a 95% confidence interval on each PWM. After applying the Bonferroni correction for multiple hypotheses, no factors remained significant (Figure 6, 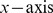).

Second, we hypothesized that LNCaP cell-specific enhancer regions might differ from random SNPs in the relative abundance of some motifs, and therefore might be a more appropriate background. To test this, we repeated the procedure of random selection of SNPs, this time filtering by the same genomic regions used in our *Funci*{*SNP*} analysis to define putative enhancers. Figure 6 shows the relationship of the estimates to random background *vs.* random draws from LNCaP biofeatures. To make the results comparable between different motifs, we expressed the observed motif disruptions as a 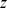 statistic. This statistic is a ratio of the difference in counts of disrupted motifs from the mean to the standard deviation (see Methods, eq. 2). None of the factors of special interest in prostate cancer, *i.e.* MYC, FOXA, AR, GATA1 or 3, ETS1, TCF7L2, and NKX3-1, were enriched compared to LNCaP background. The regression line (in blue) clearly indicated significant deviation from the line of unity, suggesting greater similarity of the GWAS correlated SNPs to random LNCaP biofeature SNPs compared to background, consistent with our hypothesis. A Shapiro-Wilk test for normality revealed that the 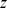 scores from LNCaP and random background are normally distributed (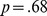 and 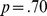 respectively). Hence, the observed deviations were largely within the range of what we expected given a random sample of SNPs in LNCaP-specific biofeatures.

### Characterization of putative target genes

Prostate cancer is driven by androgen receptor signaling [12], and is likely also influenced by basic cellular processes that contribute to other cancers [35], [36]. Therefore there are two classes of potential targets. The first is the nearest gene(s) to the risk lesion, the exact location of which is somewhat uncertain but lies in a region of probability with a local maximum at the index-SNP. In this category there are known oncogenes and tumor suppressors. The second class, which does not exclude the first, comprises genes that are known targets of regulation by the androgen receptor.

**Figure 7:**
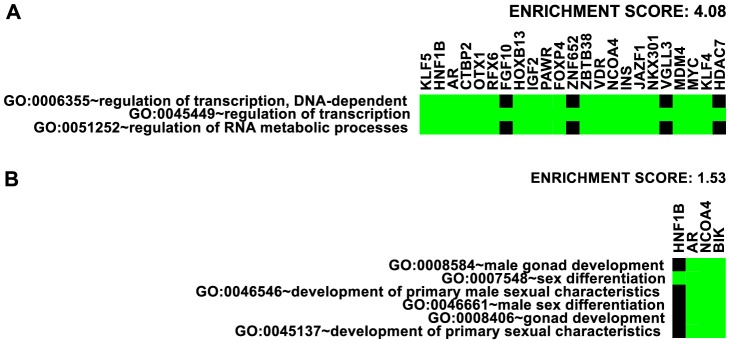
Enrichment of Gene Ontology. Representative ontology clusters from DAVID [37] enrichment analysis of nearby genes given in Table 1. Green boxes indicate membership of the genes (as columns) with the annotations (as rows). A. Transcription factor cluster. B. Male gonad development cluster.

We first took an inventory of nearby genes to the 77 risk loci (see Table 1) and analyzed gene ontology enrichment using the annotation clustering tool at the DAVID bioinformatics site [37]. The highest enrichment was for transcription factors (enrichment score 4.08, Figure 7A). Overall, 20 DNA-binding transcription factors are directly associated with 35 out of 77 independent prostate cancer GWAS loci: HNF1B, AR, CTBP2, RFX6, OTX1, HOXB13, PAWR, FOXP4, ZNF652, ZBTB38, VDR, NCOA4, JAZF1, NKX3-1, VGLL3, MDM4, MYC, KLF4, KLF5 and HDAC7. By inspection, we also identified at least 10 additional transcription factors within 500 kb of 9 other GWAS loci, that are also reasonable candidates for contributing to prostate cancer risk: SOX13, ZFP36L2, ATOH8, DLX1 & DLX2 (same locus), GATA2, SKIL, SP8, ASCL2, and DPF1. Enrichment of broader categories of genes including transcriptional regulation (enrichment score 3.44), negative regulation of transcription (enrichment score 2.52), transcription and RNA metabolism (enrichment score 2.06), nuclear compartment annotations (enrichment score 2.00), and zinc-finger proteins (enrichment score 1.46) was observed.

We also detected enrichment for genes involved in male gonad and sex differentiation (enrichment score 1.53, Figure 7B) and gland development and branching morphogenesis clusters (enrichment score 1.40). The DAVID website suggests 1.3 as an approximation for an equivalent of the group non-log 0.05 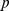 value cutoff [38]. These findings suggest that genes involved in the regulation of transcription and the differentiation of male gonad structures may be overrepresented in genomic regions with heightened risk for prostate cancer.

In our second analysis we selected all nearby androgen regulated genes within 500 kb of putative functional variants. There were 36 androgen regulated genes near 18 independent risk loci, including several from the list of transcription factors discussed in the previous section: MYC, GATA2, NCOA4, ZBTB38, ZNF652, NKX3-1. Other non-transcription factor genes were notable for being both androgen regulated and among the nearest in proximity to the GWAS hit, including KLK3 (otherwise known as prostate serum antigen [PSA]), IGF2R, CHMP2B, BMPR1B, and the cell cycle reglator Cyclin D1 (CCND1). Table 4 lists the genes and their relative expression in androgen-stimulated LNCaP cells.

**Table 4:** Androgen-regulated genes.

index SNP	Gene	100 nM DHT [63]	10 nM DHT [64]	1 nM 1881 [65]
rs10187424	ST3GAL5	−5.10		
rs7584330, rs2292884	MLPHLRRFIP1RAMP1	+6.19+1.49−1.52		
rs17181170, rs7629490rs9284813, rs2660753	CHMP2B	+2.49		
rs10934853	GATA2SEC61A1	−3.94+1.41		
rs17021918, rs12500426	BMPR1B**PDLIM5**SMARCAD1	+1.85**+1.97**+1.68		**+2.07**
rs9364554	IGF2R	+1.35		
rs6465657	ASNSBAIAP2L1BRI3BHLHA15	+2.83+1.39−1.90	−4.72	
rs1512268	**NKX3-1**ENTPD4	**+5.74**+1.67	**+10.9**	**+5.56**
8q24 region	MYC	−4.53		
rs12418451, rs11228565, rs10896449, rs7931342, rs7130881	CCND1	−2.20		
rs4430796, rs7501939, rs11649743	TBC1D3DDX52	−1.73+1.49		
rs11650494, rs7210100	TTLL6ATP5G1PHBCALCOCO2**ZNF652**	−5.66−3.89−1.64+1.41**+1.56**		**+1.98**
rs2735839	**KLK2** **KLK3** **KLK4** **KLKP1** **C19orf48**KLK15VSIG10L	**+7.83** **+3.40** **+1.47** **+2.13** **+1.60**−4.55−3.20	**+134** **+53.4** **+12.3** **+9.62** **+4.88**	**+18.8** **+6.94** **+2.77**
rs1327301, rs5945572, rs5945619	MAGED1	−1.53		

**Table of Index SNPs with AR regulated genes.** Genes within 1 Mb of functional SNPs. Genes are differentially expressed after exposure of LNCaP to androgen (see treatment in column header). Data are included from three different RNA-seq studies. Numbers represent fold change post-treatment. Genes identified by more than one study are indicated in bold typeface.

### GWAS correlated SNPs encoding disruptive variations in AR, FOXA1, and NKX3-1 response elements alter enhancer activity

To test the hypothesis that one or more of our putative functional polymorphisms disrupts a true transcription factor response element, we evaluated a sample of the enhancers in an *in vitro* heterologous enhancer-reporter luciferase assay in LNCaP cells. In the absence of good prior information, we could not predict the magnitude of the effect of a variant at a single nucleotide in a strong consensus binding site on enhancer activity. In order to obtain reliable inference on basal enhancer activity and response to androgen for possibly very slight changes, we eliminated other sources of variation such as plasmid preparation, batch and transfection effects. Thus, we sampled evenly over this parameter space (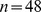) and used a hierarchical bayesian model to estimate the true enhancer activity and androgen (DHT) response, as well as the effect of SNP alleles on both (see Methods, equation 3).

**Figure 8:**
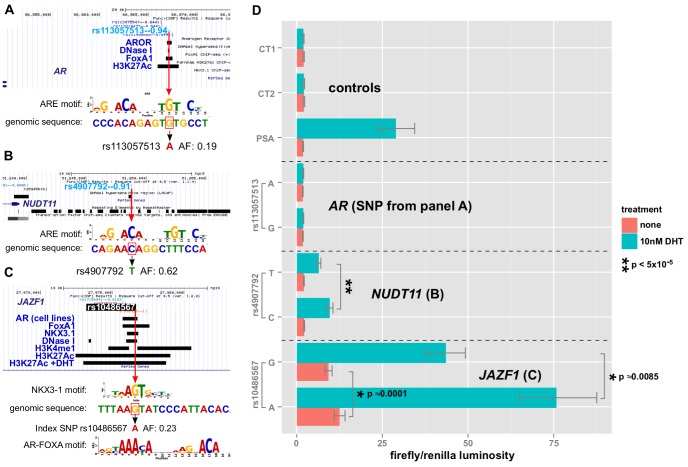
Allelic effects of prostate cancer-correlated SNPs in enhancer-luciferase assays. A,B,C: alignment of the genomic sequence surrounding the SNP with transcription factor LOGO, highlighting the disruption. Red box indicates the risk allele. Features of interest in the region are highlighted, including the biofeatures from *Funci{SNP}* analysis. D: enhancer activity in the presence or absence of DHT treatment with 95% C.I. for each allele of SNP and each enhancer (see 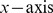 labels).

The first enhancer containing rs113057513, which encodes a consensus androgen response element (Figure 8A) near the androgen receptor gene, showed slightly elevated luciferase activity of 17.9% (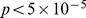) for the G allele after DHT treatment (Figure 8D). However, the difference is not biologically relevant and there was no basal activity for this enhancer relative to the negative controls.

In contrast to the enhancer at the *AR* gene locus, the enhancers near NUDT11 (Figure 8B) and in an intron of the JAZF1 transcriptional repressor gene (Figure 8C) showed a strong induction of 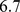
- and 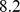-fold, respectively. Even more strikingly, both SNPs had highly significant allele specific differences in DHT-induction.

Of the three enhancers that we tested, which all contain SNPs affecting a putative ARE, the enhancer containing rs10486567 in JAZF1 showed 10-fold elevated basal activity relative to controls (Figure 8C). All three enhancers showed significantly increased activity in the presence of DHT (Figure 8D).

The NUDT11-enhancer at rs4907792 has either a T or a C allele. The C allele creates a reasonably good androgen response element by the middle C of the ACA motif, whereas the T disrupts it (see sequence logos, Figure 8B). In our luciferase assay, we did not detect a difference between alleles in basal activity, however the T allele is weaker by an estimated 1.8-fold relative to the C allele after induction with DHT. This 80% difference in the activity of the two alleles suggests that rs4907792 is critically important to the androgen sensitivity of this enhancer, and that the C allele of rs4907702 has more activity than the T allele.

For the JAZF1 enhancer, we detected a very significant difference of 1.39-fold (95% credible range of differences 1.21–1.61) in basal activity between the G and the A allele (Figure 8C, salmon bars). This particular locus is bound by the tumor suppressor NKX3-1 and the oncogene FOXA1 in LNCaP cells (Figure 8C, gbrowse view) *and* the SNP itself affects a critical residue in the response elements of both factors (see logos in Figure 8C). Thus, one version of rs10486567, encoding a G, creates a strong consensus NKX3-1 response element at this position. The alternate version of the SNP, encoding an A, destroys the NKX3-1 site in favor of an equally strong FOXA1 site.

Androgen Receptor also binds to the locus (Figure 8C) in LNCaP cells, and it is flanked by H3K4-monomethyl and H3K27-acetylation signals, providing additional evidence for this locus as a true enhancer. Consistent with a role for androgen signaling at this enhancer, we observed a 6.7-fold induction for the A allele after DHT treatment. We also detected significant allele-specific differences in DHT induction of 1.28-fold between A and G (95% credible range of differences 1.09–1.47), with the A allele being the strongest. Thus, there is an estimated mean difference of 28% in the magnitude of the androgen effect between the A and G alleles of rs10486567.

Therefore, the risk associated with the C allele of rs4907792 creates a stronger androgen response element and increased NUDT11 expression by eQTL analysis [39]. Interestingly, the risk associated with the G allele of rs10486567 in the JAZF1 intron creates an NKX3-1 binding site while destroying a FOXA1 binding site in line with the DHT-dependent decrease in enhancer activity; we would hypothesize that JAZF1 is likely a tumor suppressor influenced by this enhancer.

## Discussion

### *Funci{SNP}*

We have presented here the most comprehensive account and annotation of GWAS risk loci for prostate cancer that have been reported to date. We believe that this has value not only as a framework upon which to test new hypotheses, but to stimulate other bioinformatics efforts going forward. In the following sections we will discuss the implications of our findings with respect to the mechanisms of disease risk and the biology of human enhancers in such regions. Finally, we will explore some possible approaches for discovery of true functional SNPs by experimental means, including this work.

One of our primary motivations for using *Funci*{*SNP*} is that it restricts the number of correlated SNPs to those with biofeatures in the relevant cell type. We have chosen biofeatures associated with coding exons, microRNA regulatory targets, and most importantly, enhancers. Some loci may confer risk by alternative mechanisms, such as ncRNA, but as these are not well understood at this time, we think it best to postpone that analysis until it becomes practical. Furthermore, the vast majority of GWAS variants and their correlates lie well outside the regions where primary sequence features of that type (*i.e.* exon annotations) are present, hence we believe that many important risk variants will be identified within enhancer regions.

There are at least two other types of potential regulatory variation that are difficult to capture with this type of analysis. One is alterations to the primary sequence that, by mechanisms which have yet to be elucidated, alter the pattern of nucleosome spacing or histone modification. It is known that some sequences contribute to nucleosome positioning in chromatin [40]–[42]. A second mechanism that we have not explored in our annotation is the effect of such polymorphisms on DNA methylation at CpG sites. Such polymorphisms may contribute to variation in gene expression levels [43].

Another issue is that many identified GWAS associations consist of common variants with only slightly elevated risk (odds ratios in the range of 1.02 to 1.8 (see Figure S18). We anticipate that such small magnitude of risk is associated with very small changes in the regulation of certain key genes. Since many of the genes associated with risk loci are key regulators of development and cellular biology (*e.g.* MYC), such disruptions are necessarily tissue specific and mild so as to confer slightly elevated risk over a lifetime, and perhaps with cumulative effects or environmental interaction.

So far the vast majority of GWAS risk that has been reported does not affect protein coding regions. Indeed, as much as 77% of GWAS variation is associated with DNAse I hypersensitivity sites [44]. Our findings are consistent with this: 663 of 727 SNPs are located in enhancers. Moreover, 509 of these SNPs potentially disrupt known transcription factor response elements, *vs.* only 13 SNPs encoding putative missense mutations in proteins.

Our analysis of the missense variations in our correlated and index SNPs suggests that it is possible that a few of them encode damaging mutations, but this was by no means the unanimous conclusion from the various algorithms we tried. The only clearly damaging variant was rs138213197, which encodes a change from Glycine to Glutamate in the HOXB13 gene, and was previously reported to be associated with a high risk of prostate cancer [45]. This result was also recently confirmed in a GWAS [46]. Expression of HOXB13 is critical for mammalian prostate development [47], and likely involved in carcinogenesis of the prostate as a tumor suppressor [48], [49]. The allele frequency of this variant is very low (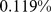), possibly suggesting lower fitness *in utero*. Furthermore the risk allele has an odds ratio of 4.42 [46] and individual carriers are likely to contract prostate cancer at an earlier age [45]. Nonetheless, it remains possible that even milder variants in one of the other proteins that we have catalogued in Table 2 also contribute to risk. It will be necessary to do follow-up allele replacement experiments either in cell lines or in other model systems, *e.g.* mouse to determine the contribution to cellular or disease phenotype, if any.

In order to zero in on which SNPs are likely to be functional and causal, we need to know which of the putative enhancer regions are most likely to be true enhancers. This information will come from a variety of sources including computational models using ENCODE data. In addition, chromatin conformation capture experiments that elucidate the intrachromosomal looping, which brings transcription factors into association with the PolII complex at promoters and thereby promotes gene transcription will be vital to this effort. ENCODE has provided some limited 5C chromatin interaction data for the MYC region, which we have superimposed on our *Funci*{*SNP*} results in Figure 4. These data show a clear relationship between the *Funci*{*SNP*} results and regions of chromatin that interact with both MYC and other genes in the region. Despite the fact that chromatin biofeatures are scattered evenly throughout the region, the correlated SNPs appear to fall only within these special regions where intramolecular chromatin interactions are apparent. It is also notable that the specialist transcription factors AR and NKX3-1 are restricted to these regions. One of the most striking examples of the power of the *Funci*{*SNP*} approach is the potentially significant information obtained for the rs188140481 index SNP, which as we have previously pointed out does not coincide with LNCaP biofeatures [50]. It resides 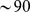 kb distant from one highly correlated SNP, rs183373024, that encodes a lesion in a strong consensus FOXA1 binding motif. Rs183373024 also resides in DNAse I and FOXA1 ChIP-seq peaks [50], as well as highly significant 5C interaction with the MYC locus (Figure 4).

Yet another clue about likely causality may be supplied by our observation that at loci where GWAS identified the same susceptibility in two or more populations, there are a subset of SNPs with greater correlation to the index in both populations. Indeed, it has been previously reported that disease associations that fail to replicate between European and East Asian populations map to regions where LD structure differs significantly [51]. Thus, the underlying LD structure has potential to inform the search for functional SNPs. Because of the importance of this point (illustrated in Figure 5), we included plots, annotated with multiethnic-significant corrSNPs, of LD structure for each region where risk was identified in more than one ethnic group in the supplementary materials. These plots should serve as a resource for followup studies being conducted on each individual region. It makes sense in our view to prioritize these SNPs over others when running empirical tests for functionality. This finding also highlights the intrinsic value of identifying the same associations in more than one ethnic group.

### On enrichment of targets

A natural question about the prostate cancer GWA studies is whether they point to specific mechanisms of risk, and whether they shed any light on the mechanisms of development of prostate cancer or cancer generally. We decided to look at the GWAS data through the lens of human genetics and to treat the set of observations the way one might approach a genetic screen in a model organism.

Since a significant fraction of the risk occurs within enhancer regions, it is a reasonable hypothesis that variations in transcription factor response elements are responsible for the majority of the functionality associated with such risk. Furthermore, if there are one or more factors whose regulatory activity in the risk regions is more important than the others, we might be able to detect enrichment in its binding site disruptions. Key to our analysis is the focus on significant disruptions, *i.e.* functional SNPs, and exclusion of SNPs that merely fall within likely binding sites. We did not find any strong evidence for enrichment of any motifs, including MYC.

An association was reported for GWAS loci LD-blocks and genome-wide androgen receptor bound regions [52]. Of course, such associations imply but do not necessitate direct involvement of the androgen receptor *per se*. We have attempted to address the association specifically with AR by selecting variants with response element disruptions. Although we did not see enrichment, we reported two SNPs that exhibit clear effects on androgen sensitive enhancer activity. However only one of the SNPs disrupts an androgen receptor response element directly. One explanation to reconcile our lack of enrichment with the previous study is that GWAS loci are indeed enriched in androgen sensitive enhancers (*i.e.* androgen bound), but the causal variants aren't biased toward disruption of a particular factor. Thus, any factor that disrupts the activity of a particular androgen-sensitive enhancer might be suspect. Biologically this makes some sense, since we expect the target gene to be more important than components of the regulatory network. It has long been known that transcription factor motifs cluster in regulatory regions [53]–[55], and it was reported recently that transcription factors cluster tightly in DNase accessible regions in a cohesin-dependent fashion [56]. This arrangement of transcription factors on enhancers *in vivo* is consistent with this latter observation. Finally, we note that even enrichment for androgen-bound mechanisms does not preclude a subset of loci having androgen-independent risk.

**Figure 9:**
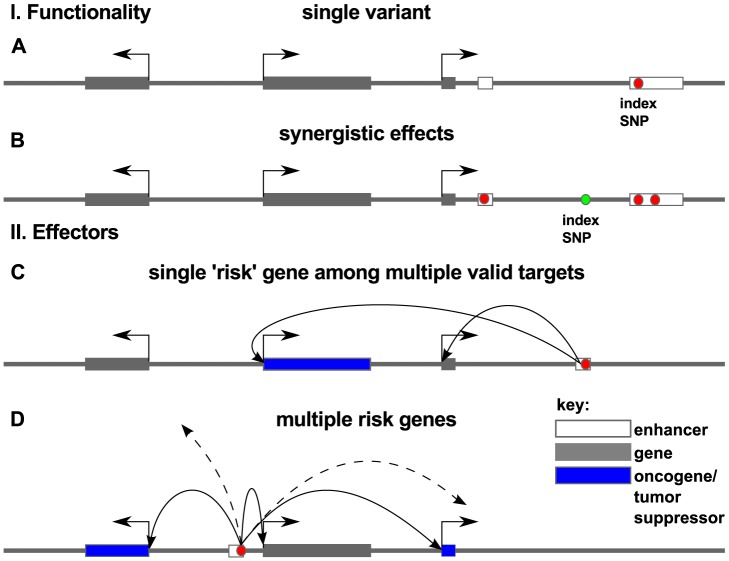
Models for association of risk with effector genes. Red dots indicate the true causal variant position in the genome, as opposed to variants that may be merely correlated with such functional variants (green dots). In panel I. we consider functionality of such variation within a locus. Causal association with risk for disease may be the result of a single variant (A) or multiple correlated variants (B) disrupting regulatory elements in enhancers (white box). In panel II we consider the effector genes of these causal variants. Arrows show regulatory interaction between enhancer and promoter as revealed by chromatin conformation capture experiments. Risk may arise from a damaging hit to a regulatory region that affects the expression of a single key oncogene or tumor suppressor (blue box) (C) or several effector genes that target a disease process or pathway (D).

It is worth mentioning the reasons we did not see enrichment and implications of this for the risk mechanism. A trivial explanation for lack of enrichment is insufficient sample size (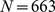). Typical disruptions for a given PWM fall somewhere in the range of 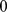 to 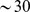 for this sample size, with a median of 6. However, a more likely scenario is that the signal is lost in the noise. If one or two SNPs carries the majority of risk (as in Figure 9A), then *Funci*{*SNP*} identifies these SNPs plus a handful of false positives. We would more likely detect true enrichment if we restricted our analysis to the set of true causal risk SNPs. On the other hand, it is possible that clouds of functional variants in correlation with the index (as in Figure 9B) carry the risk. Indeed, conserved clusters of individual transcription factor motifs are found near target genes [57]. In that case, we might have detected enrichment more readily in our correlated set even if we are capturing only some of the causal variants. Another possibility that has been proposed is that the index-SNP is loosely correlated with multiple rare, high-effect variants (the synthetic hypothesis) [58], [59], and our analysis would be insensitive to such a mechanism.

Which mechanism is most consistent with the aggregate of PCa GWAS data? We identified several regions with a large number of associated variants, for example the variants in the 8q24 region and rs7584330 (see also Figure S5). In contrast to this we also identified many examples with no variants (beside the index-SNP), including rs721048, rs1287748, rs1529276, rs4775302, rs138213197, rs11650494 and rs103294 among others. The remainder fall somewhere between these extremes. Thus, a careful review of the 77 loci suggests that a mixture of mechanisms are in play, and this alone may account for the lack of enrichment.

It is also worth considering possible underlying causes of risk. We looked at target enrichment, and found that transcription factors are enriched in the vicinity of prostate cancer risk regions. This suggests that risk is heavily influenced by perturbations to transcriptional networks. We also uncovered evidence for enrichment of factors involved in the development of male gonad and glandular structures near GWAS risk loci, all consistent with the biology of the tissue of origin for this cancer. Thus it appears that dysregulation of these genes may contribute to risk for disease.

The simplest model for risk effectors is that a causal risk SNP(s) affect the tissue-specific expression of a single key effector gene (as in Figure 9C). There is some recent evidence from GWAS in hypertension that multiple genes can be targeted [60] consistent with the model in Figure 9D in which a single GWAS hit affects multiple genes. Again, we see examples of loci that appear consistent with either model (multiple- or single-hit risk), and it will be intriguing in the coming years to uncover the true functional SNPs and their effector genes.

### Mechanisms for the effect of single nucleotide substitutions on enhancer activity

We have characterized two SNPs, rs4907792 and rs10486567, with highly significant effects in a heterologous reporter assay. These SNPs affect response elements of factors widely thought to be drivers in the progression of prostate cancer. It is interesting to compare and contrast the different effects we observed for the SNPs.

Rs4907792, which is located in the enhancer near NUDT11, directly changes a computationally identified AR response element. We observed little basal activity for this enhancer, but a 7.8-fold activation in response to DHT. We detected an 80% difference in the level of activation between the two alternate versions of the SNP, consistent with our hypothesis that the SNP itself affects a critical residue in a true androgen receptor response element.

The SNP at rs4907792 is in linkage disequilibrium with index SNPs rs5945572 (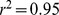) and rs1327301 (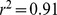), and also with index SNP rs5945619 (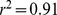), which is an eQTL with the NUDT11 gene [39]. The ‘C’ allele of rs4907792, which resulted in increased expression of reporter, correlates with the risk ‘C’ allele of rs5945619 (‘G’ in [39], referencing the bottom strand) which is associated with higher expression of NUDT11. Thus, rs4907792 is potentially the cause of slightly elevated expression of NUDT11. The eQTLs do not measure androgen sensitivity directly, and thus potentially underestimate the importance of this relationship.

In contrast, the JAZF1 enhancer that contains the index SNP rs10486567, surprisingly affects alternately good NKX3-1 or FOXA1 binding sites (see sequence logos in Figure 8C). For this enhancer we detected significant basal activity of 11 times that of the control enhancers, and also 6.7-fold activation in response to DHT. We detected an allele-specific difference in this enhancer of 28%, though significantly smaller than the NUDT11 enhancer.

These observations are consistent with rs10486567 having a direct effect on the basal transcription of the JAZF1 enhancer by altering the stoichiometric balance between FoxA1 binding and NKX3-1 binding, and an indirect but biologically relevant effect on androgen sensitivity through the androgen receptor, whose binding is promoted by FOXA1 [61].

The JAZF1 enhancer is situated in intron 3 of JAZF1, making JAZF1 the likeliest target. Consistent with our hypothesis that the index SNP rs10486567 (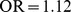) is the most significant functional variant, fine-mapping of the *JAZF1* locus suggests that this index SNP remains the most significant association in the region [62]. JAZF1 encodes a transcriptional repressor, but its expression is not regulated by androgens, at least not in LNCaP [63]–[65]. It is notable however that LNCaP is homozygous for the risk-allele ‘G’, which we found to be 39% less active and 28% less responsive to androgen. Thus, the negative result in androgen sensitive expression profiling may reflect reduced contribution of this enhancer within the regulatory milieu of LNCaP cells. Intriguingly, endometrial stromal sarcomas frequently involve rearrangements of the JAZF1 locus [66], [67]. JAZF1 may encode a tumor suppressor since loss of expression is associated with neoplastic development in multiple tumor types involving these translocations [66], though the mechanism of protective activity is unknown.

There are also two other nearby androgen regulated genes at the *JAZF1* locus, *HIBADH* and *TAX1BP1*. *HIBADH* encodes a mitochondrial enzyme, and is negatively regulated by androgen [63]. However, it is not associated with prostate development or cancer. TAX1BP1 is a likely essential inhibitor of apoptosis pathways mediated by NF-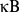 and JNK signaling [68]. Since the simplest hypothesis would involve overexpression of this gene, it is difficult to reconcile the risk allele leading to loss of TAX1BP1. JAZF1 and TAX1BP1 abut at their 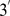 ends, so another possibility is that decreased transcription of the JAZF1 locus alters the rate of transcription or termination from TAX1BP1, thus increasing its expression and indirectly promoting the anti-apoptotic pathway.

## Conclusion

Our data and subsequent analyses paint a picture of prostate cancer risk loci in which the majority of variants overlap likely enhancer regions. But we also find a high degree of heterogeneity in the arrangement of these loci and the number and types of functional SNPs associated with them. We provided a complete summary of the functional variants associated with GWAS risk in prostate cancer, and analyzed the putative causal variants and effector genes with respect to biological enrichment. In light of these various observations, we explored the implications for mechanisms of risk, and found that our data are consistent with GWAS risk loci encoding one or more damaging variants in stage- and tissue-specific enhancers. As a preliminary step toward characterizing these variants, we cloned 3 enhancers and tested them in an enhancer-luciferase assay with different versions of the risk-associated SNPs. Two of the enhancers exhibited androgen-responsiveness, and also exhibited allele-specific differences. Therefore, it will be interesting to see whether some of the 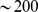 enhancers we have characterized are tissue- or stage-specific, which genes are modulated by their activity, and whether those genes in turn have an effect on cellular phenotype. Going forward, it will be necessary to characterize the effect of all the risk elements and the correlated variants on gene regulation in LNCaP. It will also be instructive to perform chromatin conformation capture experiments, to further characterize and verify the interaction of these enhancers with their target genes. As a practical concern, we have identified a seemingly large number of putative functional variants in need of testing (509 SNPs in enhancers and 20 SNPs in promoters). Once the enhancers have been tested for biological activity *in vivo* using knockout by TALen or CRISPR, the number of variants will be further reduced. These variants should then be prioritized by 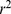, including multi-ethnic comparisons where possible, then by response element (*e.g.* an AR binding site
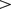
GFI1). This work will pay dividends not only for understanding the etiology of prostate cancer and similar diseases, but promises to greatly expand our understanding of the biology of non-coding sequences in the genome.

## Materials and Methods

### Genome-wide ChIP-seq

LNCaP cells were cultured as described previously [7]. For H3K27Ac experiments they were first grown with charcoal-stripped serum and harvested when 80% confluent. LNCaP were stimulated for 4 hours either with 10 nM DHT or ethanol vehicle control before collection. LNCaP for TCF7L2 ChIP-seq was grown in RPMI 1640 supplemented with 5% FBS (not charcoal-stripped) and collected when 80–90% confluent. Antibodies used for ChIP-seq were: TCF7L2 (Cell Signaling Technology, Danvers, MA, USA; C48H11 #2569, lot2), H3K27Ac (Active Motif, Carlsbad, CA, USA; #39133, Lot#213110044). For the TCF7L2 ChIP-seq assay, 835 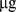 of chromatin was incubated with 25 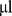 antibody; for H3K27Ac, 10 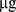 chromatin was incubated with 6 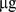 antibody. TCF7L2 and the H3K27Ac ChIP assays were performed as described [69] using protein A/G magnetic beads to collect the immunoprecipitates. Enrichment of ChIP targets was confirmed by qPCR and libraries were created as previously described [69]. Gel size selection of the 200 to 500 bp fraction was conducted after an adapter ligation step, followed by 15 amplification cycles. The TCF7L2 library was run on an Illumina GAIIx and mapped to the UCSC human genome assembly HG19 using Illumina eland pipeline. LNCaP H3K27Ac libraries were barcoded and sequenced by the University of Southern California Epigenome Center on an Illumina Hi-seq and aligned to the UCSC human genome HG19 using Bowtie 2 [70]. Peaks were called using Sole-search [71] (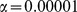, FDR 0.0001 and a blur length set to 1200 for H3K27Ac; 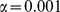, FDR 0.001 for TCF7L2). The complete data for 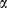-H3K27Ac ChIP-seq and 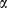-TCF7L2 ChIP-seq are deposited at GEO accession # GSE51621 (http://www.ncbi.nlm.nih.gov/geo/).

### Luciferase enhancer assays and site-directed mutagenesis

Enhancers were amplified by polymerase-chain-reaction using primers listed in Table 5 from LNCaP genomic DNA and cloned into TK-luc2 plasmid as previously described [7]. Luciferase enhancer assays and site-directed mutagenesis were performed using previously published methods [7].

**Table 5:** Primer sequences.

enhancer name	sequence	*T_m_*	prod. size
8q24 CT1	F: 5′ GGGGTACCCCAAGTGGAACCAACTGAC 3′R: 5′ GGGGTACCGGCCAAAAGAAAATGGCATA 3′	60°C60°C	1,691
8q24 CT2	F: 5′ GGGGTACCGCATGCATTAGGGGAGAAAA 3′R: 5′ GGGGTACCGTAGCTCACAGCCGAGATCC 3′	60°C60°C	1,582
AR	F: 5′ GGGGTACCCCCCCTGGTAGGTTTAGCTC 3′R: 5′ TCCCCGCGGGGCTCTTGACTTCCCTACCC 3′	60°C60°C	989
NUDT11	F: 5′ GGGGTACCTGATGAGAACACCCCACAAA 3′R: 5′ TCCCCGCGGGGCCCTGAAACAGCAATTAT 3′	60°C59°C	1,045
JAZF1	F: 5′ GGGGTACCTGCACAAACTCAGGGACAAA 3′R: 5′ TCCCCGCGGACAGCCTGATGGAGGAGCTA 3′	60°C60°C	798

**Primers used in cloning enhancers for reporter assays.** The underlined portion highlights the 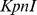 and 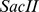 sites used for site-directed cloning of the PCR product. The PSA control is described in [7].

### Models and computation

#### *Funci*{*SNP*} analysis and assessment of SNP effects

To integrate chromatin biofeature annotations with 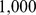 genomes genotyping data, we used in-house developed R package *Funci*{*SNP*}, available at Bioconductor.org [2]. We selected publicly available datasets that are relevant to the biology of prostate epithelia and prostate cancer. The following ENCODE datasets were employed to filter correlated SNPs that lie within putative enhancer regions with Gene Expression Omnibus (GEO) accession IDs 1) LNCaP and RWPEI DnaseI HS sites (GSE32970); PrEC DNaseI HS sites (GSE29692); LNCaP CTCF ChIP-seq peaks (GSE33213); LNCaP H3K4me3 and H3K4me1 histone modification ChIP-seq peaks GSE27823); FoxA1 ChIP-seq peaks (GSM699634 & GSM699635); Androgen Receptor ChIP-seq peaks [72] & ARBS (GSE28219 [73]); NKX3-1 ChIP-seq peaks (GSM699633). To define other physical map features (transcription start sites, 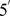 UTR, 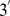 UTR) we obtained annotations from the February 2009 release of the human genome (GRCh37/HG19) in the UCSC genome browser. We used the highly conserved set of predicted targets of microRNA targeting at mircode.org (miRcode 11, June 2012 release) [18]. *Funci*{*SNP*} was run with the following settings: a window size of 1 Mb around the index SNP was used, and 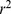 cutoff 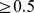. Linkage disequilibrium (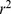) was calculated separately for all populations in which each index SNP was originally reported (see Table 1). Analysis of the potential effect of non-synonymous variants on protein folding was carried out with Provean [14], SIFT [15], Polyphen2 [16], and SNAP [17] with default settings. To determine whether *Funci–SNP}*-generated SNPs potentially affect the binding of known transcription factors, PWMs were employed from [22] and [23]. Thus the matrix score 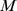 varies from 0 to 1 and is given as:
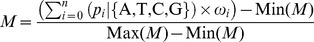
(1)where the frequency 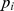 is derived from PWM of factor 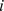 and we introduce the positional weight 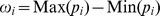 to account for the importance of the position in the motif.

#### Analysis of transcription factor response element enrichment

The 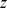 scores for motif enrichment are calculated as:
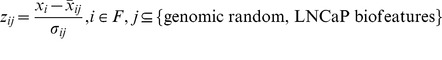
(2)where the 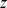 score for the 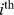 transcription factor against background 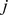 is difference of the counts 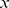 and the mean counts 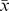 for that factor in background 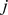, as a proportion of the standard deviation, 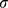. The set of transcription factors, 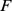, is described in the text. We calculated the bootstrapped background distribution statistics (quantiles for 2.75% and 97.5%) representing the 95% confidence interval for each PWM individually from 200 random draws of 663 SNPs from each background. A Bonferroni correction was applied to the quantiles to correct for the application of multiple hypothesis testing.

#### Bayesian model of luciferase data

We assumed 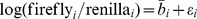 for the 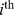 observation where the 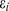, estimated from technical replication, were assumed to be exchangeable, and modeled as 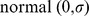 with 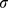 having an exponential prior with mean 1. All logarithms were natural logarithms to base 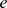. The model for the expected expression level of a given data point was
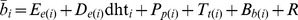
(3)where 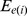 is the enhancer effect for enhancer 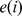, 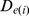 is the androgen response for enhancer 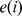, 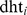 is an indicator variable for whether sample 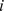 was treated with androgen hormone, 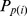 is the plasmid prep effect for plasmid prep 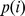, 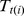 is the transfection effect for the particular transfection 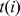, and 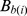 is the batch effect for all data from the 96 well plate 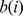. The level 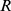 was the reference level constrained to be the average of all data for the two negative control enhancers.

There were typically 6 plasmid preps for each enhancer, and 4 transfections of each plasmid prep in each batch where that plasmid was measured. Each sample was replicated twice on the plate. The negative controls and PSA positive control were run on each batch.

The 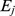 values were given a t distribution prior with degrees of freedom and scale each exponentially distributed with mean values 20, and 8 respectively. The 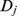 values were taken to be cauchy distributed with scale exponentially distributed with mean value 1/2. The plasmid prep effects 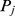 were taken to be normally distributed around 0 with standard deviation exponentially distributed with mean value 1. The transfection effects 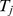 were take to be t distributed with exponential priors on degree of freedom (mean 3) and scale (mean 1/2).

Bayesian model and subsequent inferences were fitted via the Metropolis algorithm [74] using a Hamiltonian sampler implemented in Stan software [75], [76]. In the text and Figure 8, we report the mean of samples and 95% credible interval (C.I.) for contrasts of interest. We interfaced to the software via the rstan package (version 1.3.0) in the R statistical environment (version 3.0.1) on a desktop Intel i7 running Ubuntu release 12.04.

## Supporting Information

Figure S1 Histogram of H3K27Ac peaks. Peak height plotted as a function of peak number for both charcoal stripped serum (css) and DHT treatment (dht) in LNCaP cells. The dotted line indicates the cutoff top 25 k peaks used as biofeatures for *Funci*{*SNP*} analysis.
(EPS)

Figure S2 H3K27Ac Overlap of peaks +/− DHT.
(EPS)

Figure S3 Enrichment of TCF7L2 binding sites within ChIP-seq peaks. Average number of TCF7L2 motifs as a function of distance from center of peak. Red line: top 20 k peaks. Blue line: top 10 k peaks. Green line: top 5 k peaks.
(EPS)

Figure S4rs12621278 in two populations. 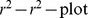 reveals SNPs that are correlated to the index SNP in both populations for which it has been identified as carrying risk. SNPs greater than 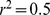 are highlighted in red.(EPS)

Figure S5rs7584330 in two populations. 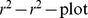 reveals SNPs that are correlated to the index SNP in both populations for which it has been identified as carrying risk. SNPs greater than 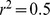 are highlighted in red.(EPS)

Figure S6rs17021918 in two populations. 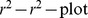 reveals SNPs that are correlated to the index SNP in both populations for which it has been identified as carrying risk. SNPs greater than 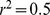 are highlighted in red.(EPS)

Figure S7rs7679673 in two populations. 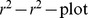 reveals SNPs that are correlated to the index SNP in both populations for which it has been identified as carrying risk. SNPs greater than 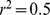 are highlighted in red.(EPS)

Figure S8rs12653946 in two populations. 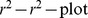 reveals SNPs that are correlated to the index SNP in both populations for which it has been identified as carrying risk. SNPs greater than 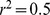 are highlighted in red.(EPS)

Figure S9rs1983891 in two populations. 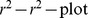 reveals SNPs that are correlated to the index SNP in both populations for which it has been identified as carrying risk. SNPs greater than 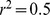 are highlighted in red.(EPS)

Figure S10rs339331 in two populations. 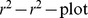 reveals SNPs that are correlated to the index SNP in both populations for which it has been identified as carrying risk. SNPs greater than 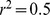 are highlighted in red.(EPS)

Figure S11rs9364554 in two populations. 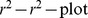 reveals SNPs that are correlated to the index SNP in both populations for which it has been identified as carrying risk. SNPs greater than 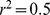 are highlighted in red.(EPS)

Figure S12rs10486567 in two populations. 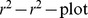 reveals SNPs that are correlated to the index SNP in both populations for which it has been identified as carrying risk. SNPs greater than 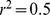 are highlighted in red.(EPS)

Figure S13rs6983267 in two populations. 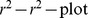 reveals SNPs that are correlated to the index SNP in both populations for which it has been identified as carrying risk. SNPs greater than 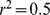 are highlighted in red.(EPS)

Figure S14rs7127900 in two populations. 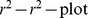 reveals SNPs that are correlated to the index SNP in both populations for which it has been identified as carrying risk. SNPs greater than 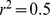 are highlighted in red.(EPS)

Figure S15rs10896449 in two populations. 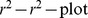 reveals SNPs that are correlated to the index SNP in both populations for which it has been identified as carrying risk. SNPs greater than 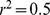 are highlighted in red.(EPS)

Figure S16rs11228565 in two populations. 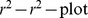 reveals SNPs that are correlated to the index SNP in both populations for which it has been identified as carrying risk. SNPs greater than 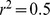 are highlighted in red.(EPS)

Figure S17rs8102476 in two populations. 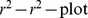 reveals SNPs that are correlated to the index SNP in both populations for which it has been identified as carrying risk. SNPs greater than 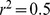 are highlighted in red. Note the vertical scale has been optimized to make the SNP label readable.(EPS)

Figure S18 Comparison of relative risk in different cancers. GWAS odds ratios of SNPs reported for various cancers for comparison with prostate cancer (red).
(EPS)
